# Multiple Infections with Viruses of the Family *Tymoviridae* in Czech Grapevines

**DOI:** 10.3390/v16030343

**Published:** 2024-02-23

**Authors:** Marcela Komínková, Karima Ben Mansour, Petr Komínek, Jana Brožová, Radomíra Střalková

**Affiliations:** 1 Ecology, Diagnostics and Genetic Resources of Agriculturally Important Viruses, Fungi and Phytoplasmas, Crop Research Institute, Drnovská 507, 161 06 Prague, Czech Republic; kominkova@vurv.cz (M.K.); or karina79@hotmail.fr (K.B.M.); brozova@vurv.cz (J.B.); 2 Department of Plant Protection, Faculty of Agrobiology, Food and Natural Resources, Czech University of Life Sciences Prague, Kamýcká 129, 165 00 Prague, Czech Republic; 3 Crop Research Institute, Prague, Research Station for Viticulture Karlštejn, Karlštejn 98, 267 18 Karlštejn, Czech Republic; stralkova@vurv.cz

**Keywords:** high-throughput sequencing, phylogenetic analysis, grapevine fleck virus, grapevine rupestris vein feathering virus, grapevine red globe virus, grapevine syrah virus 1, grapevine asteroid mosaic-associated virus

## Abstract

This study focused on the viruses of the *Tymoviridae* family that infect grapevines in the Czech Republic. Complete sequences of GFkV (grapevine fleck virus) and GRGV (grapevine red globe virus) from the genus *Maculavirus* and GRVFV (grapevine rupestris vein feathering virus) and GSyV-1 (grapevine Syrah virus 1) from the genus *Marafivirus* were obtained using high-throughput sequencing of small RNAs and total RNAs. Mixed infections with these viruses were observed, as well as several variants of these viruses in the same plant. Phylogenetic analysis showed the position of the newly obtained virus isolates within the *Tymoviridae* family. Recombinant analysis provided evidence of single and multiple intraspecific recombinations in GRGV, GSyV-1, and GRVFV. Additionally, GAMaV, a grapevine virus from the genus *Marafivirus*, was reported for the first time in the Czech Republic.

## 1. Introduction

Grapevine (*Vitis vinifera* L.) is a well-established cultivated perennial crop of great economic importance worldwide. Due to its vegetative propagation and multiplication, more than 100 viruses of different taxonomic groups have accumulated in grapevine genotypes during thousands of years of cultivation [1]. Many reports on the grapevine virome have been published worldwide [2,3,4,5,6] and including reports from the Czech Republic [7]. Thus, many viruses from different families infecting grapevines, including viruses from the *Betaflexiviridae* family, have been reported in the Czech Republic. These include grapevine pinot gris virus (GPGV), a member of the *Trichovirus* genus [8], as well as grapevine virus A (GVA) and grapevine virus B (GVB), members of the *Vitivirus* genus. Additionally, grapevine rupestris stem pitting-associated virus-1 (GRSPaV-1), a *Foveavirus*, has also been reported. From the family *Secoviridae*, grapevine fanleaf virus (GFLV), and Arabis mosaic virus (ArMV), both members of the *Nepovirus* genus, and from the family *Closteroviridae*, and grapevine leafroll-associated virus 1 (GLRaV-1) and grapevine leafroll-associated virus 3 (GLRaV-3), members of the *Ampelovirus* genus, have been reported in the Czech Republic [9]. In the present study, we specifically focused on grapevine viruses belonging to the *Tymoviridae* family. Two genera of the *Tymoviridae* family contain viruses that infect grapevines: *Maculavirus* and *Marafivirus*.

Two viruses belonging to the *Maculavirus* genus have been identified in the Czech Republic: grapevine fleck virus (GFkV), which belongs to the *Maculavirus vitis* species (the binomial nomenclature used for virus taxonomy by the International Committee on Taxonomy of Viruses [10,11]), has a worldwide distribution [12,13,14] and was first reported in the Czech Republic by serological means [15]. The virus is phloem restricted [16]. In young leaves of *Vitis rupestris* Scheele, the virus causes the clearing of the veinlets, which are stripped of color. The virus is latent in *Vitis vinifera* L. [17]. It does not have a known vector but can be transmitted via grafting and dispersed by the exchange of grapevine propagation materials. The second maculavirus is the grapevine red globe virus (GRGV), which also induces specific symptoms in *Vitis rupestris* Scheele but does not cause any symptoms in *Vitis vinifera* L. [18,19]. The virus was first reported in grapevines from Italy and Albania [18] and has been documented in many other European [5,20,21,22,23,24,25] and non-European countries (USA [20], China [26], Brazil [6], Iran [27], Japan [28], and Australia [29]). GRGV was first detected in Czech siRNA data during a comparative study on the efficiency of different bioinformatic pipelines in 2019. Only a small genomic fragment was obtained from this study due to the limited availability of complete GRGV sequences in the NCBI database [30].

The *Marafivirus* genus of the *Tymoviridae* family has two viruses: Grapevine rupestris vein feathering virus (GRVFV), which was first reported in Greece [20] and has since been identified in many grapevine-growing regions worldwide [3,31]. In 2016, it was first reported in the Czech Republic through HTS and confirmed through the use of RT-PCR [7]. The virus causes mild asteroid symptoms in *Vitis vinifera* L. and vein feathering in *Vitis rupestris* Scheele [20]. The second marafivirus is the grapevine syrah virus 1 (GSyV-1), from the species *Marafivirus syrahensis*, first reported in the USA on *Vitis vinifera* cv Syrah [32]. Since then, it has been found in Chile [33], Italy [34], Hungary [35], South Africa [36], China [37], Croatia [38], Spain [39], Korea [40], Russia [41], and many other countries. The virus was reported in the Czech Republic and Slovakia after an investigation using an improved RT-PCR protocol on grapevines collected from both countries [42]. There is limited data on the effect of GSyV-1 on grapevine production [3,38]. GSyV-1 is not limited to infecting *Vitis vinifera*, as it has also been found in wild blackberries under the name Grapevine virus Q [43].

Previously, we only reported the presence of these viruses in the Czech Republic. However, we were unable to present their full-length sequences because of the unavailability of complete reference sequences in the public databases. This is no longer the case, thanks to the widespread use of HTS for plant viruses and, in particular, for grapevine virus genomics [2,4,44], which has resulted in the presence of a large and increasing number of full-genome sequences of grapevine viruses. Therefore, our efforts were aimed at obtaining and analyzing full-length sequences of *Tymoviridae* viruses to understand the viral diversity in the Czech Republic.

## 2. Materials and Methods

### 2.1. Sample Collection and RT-PCR

Grapevine plants for the present work were obtained from the collection of plant viruses at the Crop Research Institute, Prague (collection VURV-V, part of the collection VURV, officially recognized by the World Federation for Culture Collections), from the basic and prebasic propagation material vineyards at the Research Station for Viticulture Karlštejn (which is a part of the Crop Research Institute, Prague), and from surveys conducted in the Czech viticultural regions [7,9,15]. Among the hundreds of grapevines surveyed and tested, twelve grapevines were selected for the present study based on the positive RT-PCR reaction with generic primers for the family *Tymoviridae* [18] (Table 1). None of the selected grapevines exhibited any symptoms of viral infection.

Total RNA was isolated from grapevine leaves using the Spectrum™ Plant Total RNA Kit (Sigma-Aldrich, St. Louis, MO, USA). The quality and quantity of the RNA was assessed using agarose gel electrophoresis and photometric analysis (NanoDrop; Thermo Fisher, Waltham, MA, USA). It was then subjected to RT-PCR using a One-Step RT-PCR Kit (Qiagen GmbH, Hilden, Germany) and generic primers [18]. The presence of amplicons (386 bp) indicates the presence of either maculavirus or marafivirus in the grapevines studied. The PCR products were excised from the agarose gel and purified using the Min Elute ™ Gel Extraction Kit (Qiagen GmbH, Hilden, Germany). The purified PCR products were cloned into the pGEM-T Easy plasmid (Promega, Madison, WI, USA). A minimum of ten clones were taken from each sample and sequenced commercially (Macrogen Europe, Amsterdam, The Netherlands).

### 2.2. High-Throughput Sequencing (HTS)

Based on the sequencing results of the PCR products, four grapevines (TI23, LAM3, LAM8, and BLA1) were selected for high-throughput sequencing (HTS). Ribosomal RNAs were removed from total RNA preparations of the grapevine plants using a RiboMinus Plant Kit for RNA-Seq (ThermoFisher Scientific) according to the manufacturer’s instructions. Total RNA libraries were then prepared using the TrueSeq Stranded mRNA Kit (Illumina), following its simplified protocol without poly-A RNA enrichment. They were sequenced commercially using a MiSeq instrument (Illumina) at the BIOCEV Center in Vestec, Czech Republic, for paired read sequencing (2 × 150 bp). At first, the four grapevines were sequenced together in a single MiSeq run. To obtain a higher sequencing depth, the sample BLA1 was run again as a single MiSeq run.

The total RNA from TI23 was extracted from 1 g of scraped phloem, and libraries of small RNAs were prepared and sequenced using Illumina HiScanSQ (SELGE, University of Aldo Moro, Bari, Italy) as described in [7].

### 2.3. Bioinformatic Analysis

The data obtained from the two MiSeq runs were analyzed using Geneious Prime software, version 2022.1.1. The resulting reads were trimmed using the BBduk trimmer embedded in Geneious. Duplicate reads were removed, and the remaining reads were paired and merged. De novo assembly was performed using Geneious assembler with default parameters and a sensitivity set to medium/fast. De novo contigs were then annotated using the BLAST module of Geneious to identify homology to a local database of viruses and viroids downloaded from the NCBI RefSeq database (https://www.ncbi.nlm.nih.gov/refseq/ accessed on 20 March 2023).

Contigs identified as members of the *Tymoviridae* family were checked using the NCBI’s online BLAST tool to find the closest full-length sequences based on E-value. Matching GenBank sequences were then used to map the HTS reads. Mapping was performed using the Geneious mapper with the following parameters: high sensitivity/medium and a minimum mapping quality of 20. Supplementary Table S1 contains a list of the used reference sequences.

The dataset from the TI23 plant obtained through siRNA sequencing [7] was used to map and assemble GRGV, GRVFV, and GSyV-1 genomes. In addition to the Geneious mapper, sequencing data were uploaded to the Galaxy web platform using the public server at usegalaxy.org [45]. The BWA tool was used to map the siRNA reads using the default parameters. For the mapping and assembly of siRNA reads for a given virus, we employed only unused reads from the mapping of a second sequence of the same virus from the same plant/dataset.

Newly acquired full-length sequences were analyzed for their genomic structure. ORFs were identified using NCBI’s ORF Finder online tool (https://www.ncbi.nlm.nih.gov/orffinder/ accessed on 1 November 2023). Conserved domains were identified using the NCBI Conserved Domains Search online tool (https://www.ncbi.nlm.nih.gov/Structure/cdd/wrpsb.cgi accessed on 1 November 2023).

The newly obtained complete sequences were also verified through Sanger sequencing of PCR products obtained with primers specific to the respective virus variant (Table S5).

### 2.4. Recombination, Phylogenetic, and Sequence Demarcation Analyses

This report examines five viruses: GFkV, GRGV, GRVFV, GSyV-1, and GAMaV. For the recombination, phylogenetic, and sequence demarcation analyses, five datasets were created. Each dataset included the Czech isolates, the subject of our study, and the remaining isolates were retrieved from the NCBI. Therefore, each of these datasets contained a total of 42 (GFkV), 19 (GRGV), 58 (GRVFV), 30 (GSyV-1), and 10 (GAMaV) sequences (Table S3).

All datasets were aligned using the MAFFT online service [46], and then trimmed to the appropriate region, to the complete coding region for GRGV, GRVFV, and GSyV-1 and to the partial replicase for GFkV, and GAMaV, due to the unavailability of complete sequences in the NCBI database, using BioEdit 7.2.5 software [47].

RDP4 software was used to screen for any potential recombination events within the different datasets. To be considered real, the recombination event must be detected by at least four algorithms with *p*-values < 10^−6^ across the seven algorithms implemented within this software [48,49].

The MEGA X program was used to detect the best-fitted substitution model. Subsequently, the maximum-likelihood phylogenetic trees were constructed based on either the complete genome or a partial fragment of RdRp. Their phylogenies were tested with 500 bootstrap replicates. Four outliers, (KX171167, GRGV), (MN879754, citrus virus C), (MZ440710, GSyV-1), and (MZ451101, GRVFV), representing the closely related sequence to the virus of interest, were used as outliers to root the phylogenetic tree of GFkV, GRGV, GRVFV, and GSyV-1, respectively.

The Sequence Demarcation Tool (SDTv1.2) and BioEdit software were used to determine the pairwise identity and sequence similarities between the Czech isolates and other NCBI-retrieved sequences within each dataset [47,50].

## 3. Results

### 3.1. Sequencing of RT-PCR Products Obtained with Generic Primers for the Family Tymoviridae

A total of 120 fragments of the polymerase gene, originating from 12 Czech grapevines, were obtained through sequencing of the cloned RT-PCR products using generic primers for the *Tymoviridae* family. Among them, 43 sequences were unique, and five viruses belonging to the *Tymoviridae* family were identified. All tested grapevines were infected by at least one member of the *Tymoviridae* family, with GFkV being the most common virus (found in eight grapevines) and the most abundant virus in grapevine based on the number of sequenced clones per plant identified as GFkV, followed by GRVFV, which was found in seven grapevines. GRGV was detected in three grapevines, GSyV-1 was detected in two grapevines, and grapevine asteroid mosaic-associated virus (GaMaV) was detected in one grapevine (KA7). Five grapevines were infected with only one member of the family *Tymoviridae*. In addition, seven grapevines were found to be co-infected with multiple viruses (Table 2). Unique sequences obtained from the polymerase gene of *Tymoviridae* members were submitted to GenBank and are available under acc. nos. OR826216-OR826260.

### 3.2. HTS Data Analysis and Identification of Viruses

Grapevines with interesting characteristics were selected for subsequent HTS analyses. Based on the sequencing results using generic primers, two plants (LAM3 and LAM8) were found to be infected with GFkV and GRVFV. GFkV was the virus of interest in these plants, as it has only one complete sequence present in databases worldwide, without any ambiguities. Plant TI23 was selected as a positive control (internal standard for this study) because it was previously identified as a grapevine infected with several members of the *Tymoviridae* family. Additionally, plant BLA1 was selected because it contains three members of the family *Tymoviridae*, namely GRVFV, GRGV, and GSyV-1.

The MiSeq run with four total RNA libraries produced between 186,218 and 908,759 unique reads per library, resulting in the identification of seven viruses (GLRaV-1, GRSPaV-1, GVA, GVB, GPGV, GFkV, and GSyV-1) and two viroids (Hop stunt viroid (HSVd) and grapevine yellow speckle viroid 1 (GYSVd-1)). Although all four plants contained a member of the *Tymoviridae* family as detected through RT-PCR using generic primers, the HTS run allowed for the detection of a *Tymoviridae* family member in only three of the plants: BLA1 contained a mapped read of GSyV-1 and plants LAM3 and LAM8 had GFkV reads (422 and 296 reads, respectively). The library from grapevine TI23 contained 277,961 unique reads, allowing for the identification of only non-*Tymoviridae* viruses. None of the reads from this plant/library were mapped to any member of the *Tymoviridae* family, even after attempts with different parameters of the mapping algorithm or using the newly obtained *Tymoviridae* genomes as references (Table 3).

Having obtained this result, we focused on a library from the plant BLA1 and reran it as a single MiSeq run. This approach gave us 9,590,966 unique reads, allowing for higher coverage to reference sequences and the detection of three members of the *Tymoviridae* family in this single library/plant—GRGV, GRVFV, and GSyV-1. However, the genome coverage was insufficient to construct the complete genome of any of the three *Tymoviridae* members (Table 4).

Finally, we used an older HTS dataset of siRNA from the TI23 plant. It contains 19,854,724 unique reads. The presence of two tymoviridae viruses (GRVFV and GSyV-1) was reported and confirmed in a previous study [7]. Moreover, GRGV was later confirmed in the data [30]. In this work, the three previously identified viruses of the *Tymoviridae* family—GRVFV, GRGV, and GSyV-1—were successfully mapped into different reference sequences. All three viruses were present in the plant, and two genomic sequences of each virus were obtained (Table 5).

The use of two different platforms (Geneious and Galaxy) for the mapping of viruses of the family *Tymoviridae* gave slightly different results for a number of mapped reads, except for GRGV-1 and GRVFV-5, where the number of mapped reads for Geneious was almost double that for the Galaxy platform, and GSyV-1-3, where the number of mapped reads for the Galaxy platform was approximately twice that for Geneious. However, the consensus sequences from both platforms were also very similar, reaching 99% identity. The Galaxy results were used to construct the final sequences. The sequences were verified via Sanger sequencing of RT-PCR products obtained with variant-specific primers (Table S5).

### 3.3. Grapevine Fleck Virus (GFkV)

The virus was found in eight plants via polymerase region sequencing (Table 1). Using HTS, the virus was found only in LAM3 and LAM8 plants. The reads that were mapped to the GFkV reference sequence provided 92.8% (LAM3) and 75.9% (LAM8) genome coverage (Table 4). Gaps in the HTS sequence were filled via Sanger sequencing of RT-PCR products. The complete GFkV sequence from the LAM3 library was submitted to GenBank and is available under accession number OR701334. The genomic structure of the full-length GFkV sequence is typical of maculaviruses, with one large open reading frame (ORF) that encodes a polyprotein of 1949 aa and an additional ORF that encodes a coat protein of 230 aa.

Comparison of the genome of the Czech isolate (OR701334) with the Italian NCBI sequence (NC_003347), the only full-length sequence without ambiguity, showed 91.6% sequence similarity, and the viral genomes’ structure was identical. A relatively high amino acid divergence between the two sequences was observed in ORF3 (88.3%) and ORF4 (87.2%) (Table 6).

**Figure 1:**
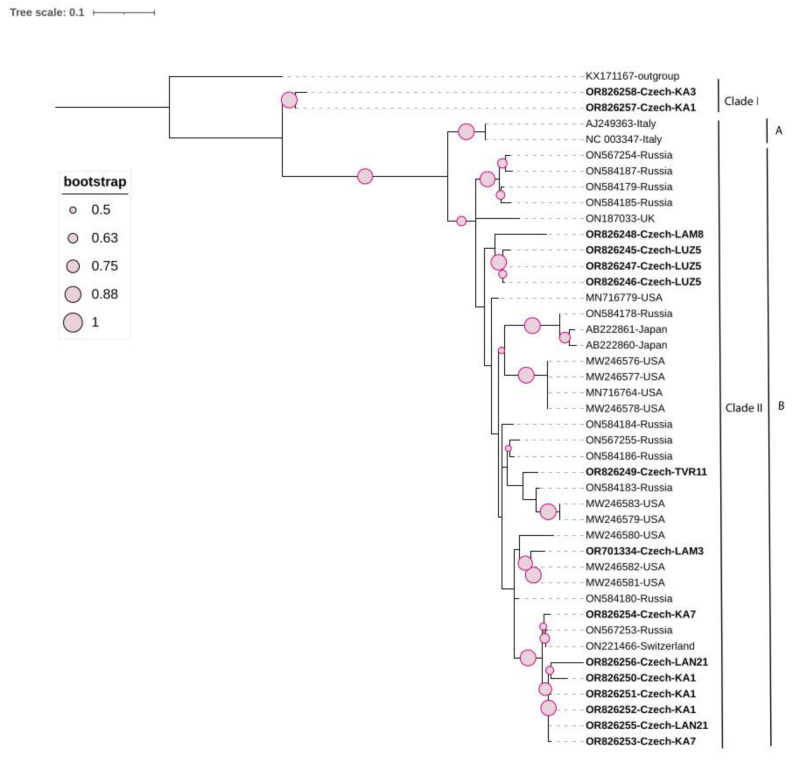
Maximum likelihood (ML) phylogenetic tree of 42 grapevine fleck virus isolates, including 15 Czech isolates (highlighted in bold), constructed based on the partial replicase fragment (342 nt). One sequence of GRGV (KX171167) was used as an outgroup. The tree was viewed using iTOL.

**Figure 2:**
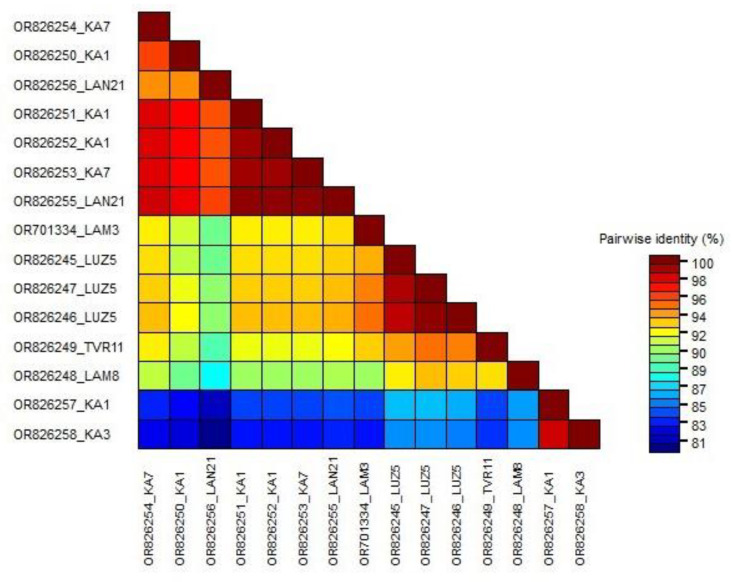
Matrix of pairwise nucleotide identity between 15 different Czech GFkV isolates calculated based on the partial fragment of replicase using SDTv1.2 software.

An ML phylogenetic tree was constructed from the partial fragment of the replicase using the best-fitted method (HKY + G + I). The tree defines two clades: Clade I contains only two isolates (OR826257 and OR826258) of Czech origin, separated from all other members assigned to Clade II (Figure 1). They shared a pairwise sequence similarity of 98.5% according to the SDT result and between 80.1–85.7% with all of the remaining isolates, members of Clade II (Figure S1). Clade II is subdivided into two sub-clades: A and B. The remaining thirteen Czech isolates clustered into Sub-clade B with members of various origins, showing high genetic diversity; three sequences (OR826245-OR826247) isolated from the same grapevine LUZ5 and one sequence (OR826248) isolated from grapevine LAM8 clustered together, with a pairwise sequence similarity of 93% between isolates from both grapevines. Two isolates (OR826249 and OR701334) recovered from grapevines TVR11 and LAM3, respectively, had a pairwise similarity of 90.6% between them, and both clustered with isolates from the USA and Russia. Seven isolates extracted from grapevines KA7, LAN21, and KA1 (OR826250-OR826256) shared a pairwise sequence similarity varying between 94.7–99.7% and clustered with isolates from Russia and Switzerland. The SDT results showed a genetic variation of 12.6% between the Czech isolates extracted from eight grapevines (Figure 2).

It is noteworthy that the sequence OR826257 and other isolates extracted from the same grapevine (KA1) did not cluster together; instead, the first sequence was a member of Clade I, whereas the other sequences clustered together within Clade II, in which both variants had 82.5–83.6% pairwise nucleotide identity.

### 3.4. Grapevine Red Globe Virus (GRGV)

Sanger sequencing of the polymerase region revealed that the virus was present in three plants (TI23, KA8, and BLA1). However, the virus was not detected in the MiSeq HTS run on multiple libraries from TI23 and BLA1, which resulted in 277,961 and 186,218 unique reads, respectively. It was only present in the MiSeq HTS run on a single library from BLA1, which resulted in a dataset containing 9,590,966 unique reads. However, the coverage of the reference sequence only reached 29.8% of its genome (Table 4). The coverage of the GRGV genome did not increase, even when the newly obtained sequences from this report were used as references for mapping.

The siRNA reads from the TI23 plant enabled the obtention of two full-length sequences: OR787584 and OR787585. They were obtained by mapping 287,329 and 352,316 reads to their respective reference sequences (Table S1). The two variants were found to be 83% identical at the nucleotide level. They contain a large ORF encoding a putative polyprotein with domains for methyltransferase, peptidase, helicase, and RNA polymerase domains. In addition, a short ORF encoding a putative coat protein is located close to the 3’ end of the genome.

Using the RDP4 program, a recombination event was identified (Table S4). It consists of a 5086 nt fragment detected in the methyltransferase, protease, helicase, RdRp, and CP. The two recombinants that share this event are (OR787585, Czech Republic) and (KX171167, Spain). The RDP4 program identified OR787584 (Czech Republic) as the major parent and MZ451074, a sequence from Canada, as the minor parent.

**Figure 3:**
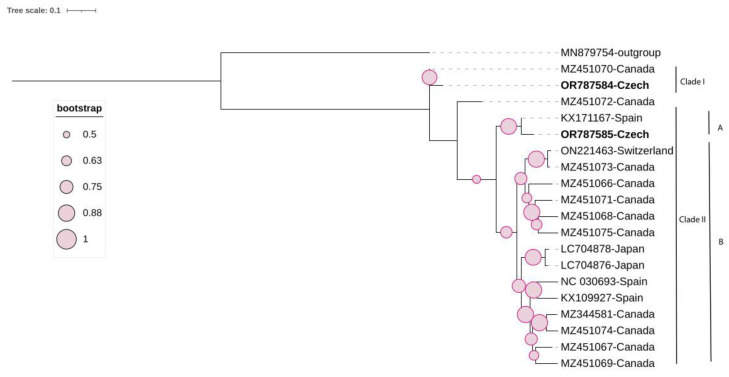
Maximum likelihood (ML) phylogenetic tree of 19 grapevine red globe virus isolates, including 2 Czech isolates (highlighted in bold), constructed based on their complete coding region. One sequence of Citrus virus C (MN879754) was used as an outgroup. The tree was viewed using iTOL.

The ML phylogenetic tree was generated with the general time-reversible model substitution model with Gamma distribution (GTR+G) based on a complete genome (Figure 3). The tree shows two clades; Clade I includes two isolates, one of Czech origin (OR787584) and the other of Canadian origin (MZ451070); they share 97% pairwise similarity (Figure S1). Clade II consists of two sub-clades, A and B. The second sequence (OR787585) isolated from the same plant (TI23) clustered with a Spanish isolate (KX171167) in Sub-clade A, sharing 97.2% pairwise similarity. Both sequences are recombinants, as previously mentioned. The two molecular variants (OR787584 and OR787585) exhibited a high degree of variation (17.5%) calculated based on the complete coding region (Figure S1).

### 3.5. Grapevine Rupestris Vein Feathering Virus (GRVFV)

Sanger sequencing of the polymerase region showed that seven out of the twelve tested plants were positive for GRVFV. Depending on the sequencing depth, the unique reads resulting from the MiSeq run on multiple libraries ranged from 186,218 to 908,759, and we could not confirm the presence of GRVFV in all of the plants (TI23, LAM3, LAM8, and BLA1). However, once the number of unique reads increased more than fifty-fold (9,590,966) following the use of an MiSeq run on a single library (BLA1), the virus could be detected. Its mapping to the reference sequence resulted in only 9.4% genomic coverage. Similarly, GRVFV was detected in grapevine (TI23) once the number of unique reads increased more than seventy-fold (19,854,724) compared to the MiSeq run on multiple libraries, allowing for 100% genomic coverage to the reference sequence. Therefore, two complete GRVFV sequences were obtained and deposited in GenBank under accession numbers OR787588 and OR787589. The sequences share 79.32% nucleotide identity. The marafiviruses share a common genome organization, which includes a large ORF that produces putative polyprotein with methyltransferase, peptidase, helicase, RNA polymerase, and coat protein domains.

The RDP4 program detected five recombination events affecting five isolates (MZ451087, MZ451088, AY706994, MN974276, and OR787588). All of the recombinant isolates, except for one isolate (OR787588) originating from the Czech Republic, were of non-European origin, (Table S4). This isolate has a 213 nt recombinant fragment detected across the replicase coding region. MZ451085 (major, Canada) and LC619667 (minor, Japan) are the detected parent for this recombination event. Based on the information available on the public database (NCBI), it appears that one or both of the predicted parents’ host plants were from non-*Vitis* host species, specifically *Prunus*.

**Figure 4:**
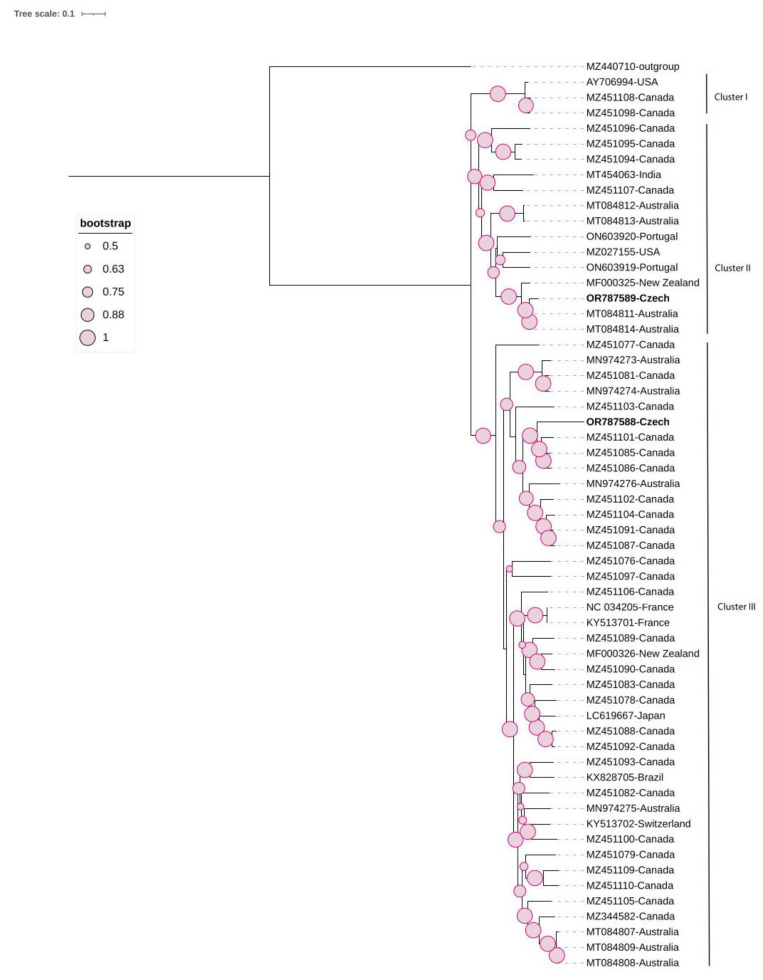
Maximum likelihood (ML) phylogenetic tree of 58 grapevine rupestris vein feathering virus isolates, including 2 Czech isolates (highlighted in bold), constructed based on their complete coding region. One sequence of GSyV-1 (MZ440710) was used as an outgroup. The tree was viewed using iTOL.

The ML phylogenetic tree was constructed using the General Time Reversible model based on the complete genome. It showed the presence of three clusters: I, II, and III. The two Czech molecular variants of GRVF clustered differently (Figure 4). The first isolate (OR787589) belonged to Cluster II, while the second isolate (OR787588) was a member of Cluster III. The pairwise nucleotide identity between both Czech molecular variants was low, at 80.5% (Figure S2).

### 3.6. Grapevine Syrah Virus-1 (GSyV-1)

**Figure 5:**
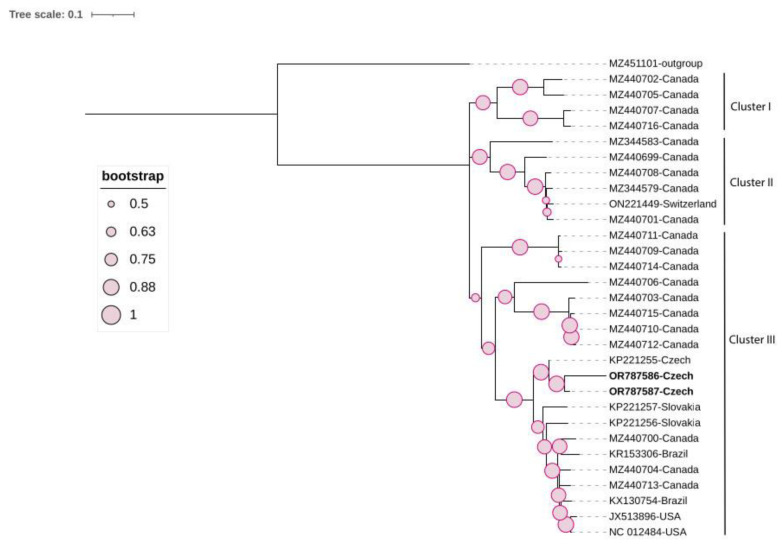
Maximum likelihood (ML) phylogenetic tree of 30 grapevine Syrah virus-1 isolates, including 2 Czech isolates (highlighted in bold), constructed based on their complete coding region. One sequence of GRVFV (MZ451101) was used as an outgroup. The tree was viewed using iTOL.

The MiSeq run on multiple libraries showed the presence of the virus in a grapevine, BLA1, where we detected one read. The result was further confirmed via MiSeq run on a single library and Sanger sequencing. Although the virus was detected in the TI23 grapevine through Sanger sequencing and siRNA HTS, no related reads were revealed through the MiSeq run on multiple libraries. Using the siRNA dataset, two full-length GSyV-1 sequences were obtained. Both isolates were deposited in GenBank under accession numbers OR787586 and OR787587. The two isolates share 93% sequence nucleotide similarity and showed a genome structure typical of marafiviruses, with a large ORF encoding a 2081 aa polyprotein containing all specific domains. The ML phylogenetic tree was constructed using Hasegawa, Kishino, and Yano substitution model and Gamma distribution (HKY+G). Figure 5 shows an ML phylogenetic tree made based on the complete genome, which was subdivided into three clusters: I, II, and III. The majority of NCBI-retrieved isolates were from Canada and were mainly grouped within Clusters I and II; only one European isolate was grouped with those sequences. Cluster III was the most diverse group, containing isolates from both the European continent (the Czech Republic and Slovakia) and the American continent (USA, Canada, and Brazil). The Czech isolates formed a separate group, sharing 91.6–93% pairwise sequence identity and 81.4–90.3% pairwise sequence identity with other groups in Cluster III (Figure S3).

Recombination analysis was performed using 30 GSyV-1 sequences, revealing that seven isolates were recombinants (Table S4). The two North American isolates (JX513896, MZ440699) had only one recombination event, while five isolates from central Europe, three Czech isolates (KP221255, OR787586, and OR787587), and two Slovak isolates (KP221256-KP221257) had multiple recombination events (n = 10).

### 3.7. Grapevine Asteroid Mosaic-Associated Virus (GAMaV)

**Figure 6:**
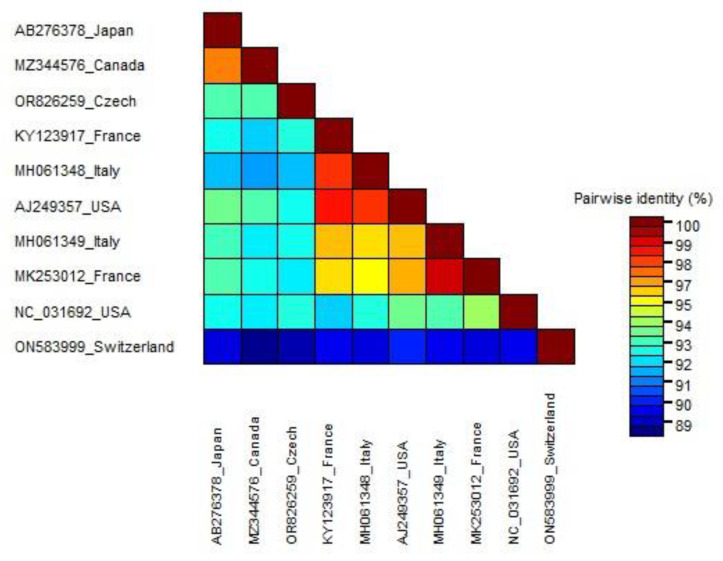
Matrix of pairwise nucleotide identity between 10 different GAMaV isolates calculated based on the partial fragment of replicase using SDTv1.2 software.

Sanger sequencing revealed the presence of the virus in only one plant, KA7. The partial replicase fragment was deposited in GenBank under accession number OR826259. The infected grapevine is a prebasic propagation material that originated from Viticulture Station Karlštejn. The SDT result indicates that the Czech isolate (OR826259) shares between 88.6 and 93% pairwise similarity with the other isolates retrieved from the NCBI (Figure 6). The Czech isolate shows the highest similarities with two isolates (MZ344576 and AB276378) from Canada and Japan (93%). All of the GAMaV isolates were divergent from the isolate from Switzerland.

## 4. Discussion

In this study, we attempted to obtain the complete sequences of the viruses of interest using HTS. We observed that the number of viruses detected in a grapevine and the genomic coverage to the reference sequence increased with the sequencing depth. This observation was confirmed by the TI23 grapevine, which served as a positive control for the approaches using cloning of RT-PCR fragments obtained with generic primers and for the MiSeq run on multiple libraries. Thus, the failure to detect the full set of viruses that were identified and confirmed in two of our previous studies [7,30] showed that these methods could give negative results, which is in agreement with an earlier report stating that sequencing depth and viral concentrations in grapevine tissues and isolated RNAs affect the successful detection of viruses [30]. Previously, the genome coverage of GRVFV and GSyV-1 was incomplete, reaching 33% and 68%, respectively [7,30]. In this study, we applied more recent bioinformatic methods and used the newly available NCBI sequences to obtain complete genomes of members of the *Tymoviridae* family. Two platforms were used for HTS analysis: Geneious and the web-based Galaxy project. Finally, we successfully assembled one complete GFkV sequence and two complete molecular variants of GRGV, GRVFV, and GSyV-1. These viruses were found in either simple or mixed infections. Co-infection (GFkV+GRVFV) was located in four grapevines (KA1, KA3, LAM3, and LAM8), and co-infection (GFkV+GAMaV) was found in one grapevine (KA7). Two grapevines (TI23 and BLA1) were infected by three viruses (GRGV, GRVFV, and GSyV-1). Only one member of the family *Tymoviridae* was found in five grapevines. The mixed infection of GFkV and GRVFV was reported previously in Spain and Slovenia [2,51]. Another viral combination (GRGV+GRVFV) was reported previously in Spain and was found to be frequent. Based on phylogenetic and pairwise sequence analyses, a second type of mixed infection, the presence of different molecular variants of the same virus in one plant, was identified. Therefore, grapevine KA1 had two variants of GFkV, and grapevine TI23 had two molecular variants of each of the following viruses: GRGV and GRVFV. This phenomenon has been observed in many other reports, such as the case of grapevine rupestris stem pitting-associated virus [52,53] and grapevine leafroll-associated virus 1 [54].

GFkV was the most frequently detected virus, found in eight out of the twelve examined plants. Our previous studies also reported its high occurrence in Czech grapevines [10]. According to the phylogenetic analysis based on the partial replicase, the GFkV isolates are divided into two clades, I and II, which is consistent with previous studies [16,55]. Additionally, the fifteen Czech isolates analyzed in this study were dispersed throughout the phylogenetic tree, indicating a high level of genetic divergence between them, consistent with previous research from Russia [3]. Moreover, a comparison between the Czech and Italian isolates revealed the highest divergence in amino acid identities to be 87.2% and 88.3% in ORF4 and ORF3, respectively. The effect of this divergence on the viral life cycle could not be speculated since, up to now, the role of these two proteins is still unknown [12,56]. The second most frequently detected virus via RT-PCR was GRVFV; the Czech isolates showed high genetic variability among them, which is consistent with another study that found Hungarian GRVFV isolates to be diverse [5]. Limited occurrence of three more viruses (GRGV, GSyV-1, and GAMaV) was observed. An interesting observation is that GAMaV, a marafivirus, that has been reported in California [32], Canada [57], Japan [53], Uruguay [58], France [59], Spain [60], Italy [61], Russia [3], and Hungary [5], is reported for the first time in the Czech Republic in this study, although it was found in only one plant (KA7). Expanding its genome sequence would be of great interest, especially since its detection in the Czech Republic came from an asymptomatic grapevine.

Recombination plays a crucial role in promoting adaptability to new hosts and changing environmental conditions [49,62]. It also helps to promote viral survival by reducing the number of deleterious mutations [63]. Intraspecific recombination is common in RNA viruses; few studies have screened for the presence of recombinants in viruses infecting grapevine. The most recent studies were on GPGV, which was found to be recombinant [64], GLRaV-3, GLRaV-4, GRSPaV, GVA, GVB, and GSyV-1 [6]. In this study, we looked for the possible presence of recombination and identified many recombinants. The marafiviruses (GRVFV and GSyV-1) had more recombination events than the maculavirus (GRGV). The first interesting observation was for GRVFV. All identified recombinants had either one or both parental sequences from a non-*Vitis* host (*Prunus*) based on the information communicated on the public database (NCBI). Although we are taking this information with caution, this primary result suggests that recombination may occur between GRVFV isolates from *Vitis* and non-*Vitis* hosts. An insect vector has been proposed as the reason for field transmission due to multiple infections of GRVFV+GRGV within the same plant [65]. The presence of recombinants with parental sequences from non-*Vitis* hosts suggests the likely involvement of polyphagous insects in the transmission of GRVFV. The second interesting observation is the evidence of multiple recombination events for GSyV-1 isolates from central Europe. This may indicate that this population is genetically diverse because the isolates recombine freely [66]. This is consistent with a study in Hungary showing that central European isolates are genetically diverse [5].

The examined grapevines were asymptomatic, even those that had multiple infections. This observation could be explained by the effect of genotypes such as TI23, infected by seven viruses, which is an interspecific grapevine rootstock Kober 125AA that is mostly asymptomatic when infected with viruses. However, after the transmission of viruses through grafting into some susceptible genotypes, like LN33, strong symptoms may appear [67].

Grapevine viruses are considered as a serious threat to grapevine yield and quality. As the plant is a perennial and vegetatively propagated crop, an important tool to control grapevine viruses is the certification of grapevine propagation material, as defined by law. However, this is not the case for most of the viruses of the Tymoviridae family that are the subject of our study. Under current European legislation, of all the tymoviruses, only GFkV is monitored in propagating material and only in rootstocks, not in varieties. The absence of symptoms in the plants included in our experiment confirms the low level of risk of these viruses for grapevine cultivation, and their inclusion in certification schemes for the propagation of grapevine varieties can hardly be expected. Nevertheless, care must be taken when exchanging grapevine genetic material, as this is the most common way of spreading grapevine viruses. The ever-increasing number of viruses described on the grapevine encourages caution on the part of those responsible for the health of the grapevine and, in particular, government organizations. Even harmless viruses, under certain conditions, may combine to produce devastating effects. For example, co-infection of ArMV and GVB in warm conditions had a lethal impact within two years [15], or the co-existence of grapevine leafroll-associated virus 3 (GLRaV-3) with a susceptible variety or with GVA can affect the observed symptomatology [68,69].

## 5. Conclusions

In our study, we found both mixed infections of different viruses of the family *Tymoviridae* and different molecular variants of the same virus within the same grapevine. We obtained full-length sequences of four grapevine viruses from the Czech Republic (GFkV, GRGV, GRVFV, and GSyV-1), and we found evidence of intraspecific recombination in our Czech GRVFV, GSyV-1 and GRGV isolates. A high divergence in ORF3 and ORF4 at the amino acid level was observed between the Czech and Italian GFkV isolates. GAMaV was found for the first time in the Czech Republic.

## Figures and Tables

**Table 1:** Grapevine plants used for the analysis of viruses from the family *Tymoviridae*.

Grapevine Plant	Cultivar	Origin	Locality
TI23	Rootstock Kober 125AA	Collection of plant viruses VURV-V	Prague–Ruzyně, district Prague-city
KA1	Müller-Thurgau, clone MT25/7	Prebasic propagation material	Karlštejn, district Beroun
KA3	Müller-Thurgau, clone MT30/34	Prebasic propagation material	Karlštejn, district Beroun
KA7	Müller-Thurgau, clone MT25/7	Prebasic propagation material	Karlštejn, district Beroun
KA8	Traminer	Basic propagation material	Karlštejn, district Beroun
LAM3	Blauer Portugieser	Vineyard survey	Lampelberg, Vrbovec, district Znojmo
LAM8	Grüner Veltliner	Vineyard survey	Lampelberg, Vrbovec, district Znojmo
BLA1	Riesling	Vineyard survey	Blatnice, district Hodonín
BLA2	Grüner Veltliner	Vineyard survey	Blatnice, district Hodonín
TVR11	Blaufränkisch	Vineyard survey	Tvrdonice, district Břeclav
LAN21	Müller-Thurgau	Vineyard survey	Lanžhot, district Břeclav
LUZ5	Sauvignon	Vineyard survey	Lužice, district Hodonín

**Table 2:** Identification and counting of viral clones from the family *Tymoviridae* using RT-PCR with generic primers.

Plant\Virus	GFkV	GRGV	GRVFV	GSyV-1	GAMaV
TI23		2	6	2	
KA1	8		2		
KA3	4		6		
KA7	8				2
KA8		10			
LAM3	5		5		
LAM8	2		8		
BLA1		5	2	3	
BLA2			10		
TVR11	10				
LAN21	10				
LUZ5	10				

**Table 3:** Results of the HTS MiSeq run with multiple libraries of total RNA.

Plant	Unique Reads in Total	Virus/Viroid Detected	Unique Reads Mapped to Virus/Viroid	Genome Coverage (%)
TI23	277,961	GVA	188	41.5
		GVB	38	23.7
		GRSPaV-1	161	61.7
		GLRaV-1	1357	98.8
		HSVd	36	100
LAM3	908,759	GFkV	422	92.8
		GRSPaV-1	952	99.99
		GYSVd-1	62	100
		HSVd	75	100
LAM8	328,278	GFkV	422	92.8
		GRSPaV-1	1135	99.9
		GYSVd-1	34	100
		HSVd	46	100
BLA1	186,218	GSyV-1	1	2.2
		GPGV	21	16.8
		GRSPaV-1	58	68.5
		GYSVd-1	66	100
		HSVd	54	100

**Table 4:** Results of the HTS MiSeq run with a single library from total RNA. The viruses highlighted in bold are members of the *Tymoviridae* family.

Plant	Unique Reads in Total	Virus/Viroid Detected	Unique Reads Assigned to Virus	Genome Coverage (%)
**BLA1**	9,590,966	**GRGV**	41	29.8
		**GRVFV**	9	9.4
		**GSyV-1**	47	32.3
		GPGV	265	81.8
		GRSPaV-1	1705	99.7
		GYSVd-1	293	100
		HSVd	670	100

**Table 5:** Results of mapping and obtaining full-length sequences of viruses from the family *Tymoviridae* from the siRNA library of the TI23 plant, with 19,854,724 total unique reads.

Virus Isolate	GenBank Acc. No.	Sequence Length	Reference Sequence for Mapping	Reference Length	Pairwise Nucleotide Identity with Reference Sequence (%)	Coverage of Reference Sequence (%)	No. of Reads Mapped to the Reference Sequence Using Galaxy	No. of Reads Mapped to the Reference Sequence Using Geneious
GRGV-1	OR787584	6849	MZ451070	6850	96.92	100.00	287,329	502,284
GRGV-2	OR787585	6837	KX171167	6851	97.18	100.00	352,316	413,327
GSyV-1-3	OR787586	6481	FJ436028	6506	87.09	100.00	392,806	209,449
GSyV-1-4	OR787587	6482	KP221255	6482	96.7	100.00	383,796	311,300
GRVFV-5	OR787588	6588	MZ451085	6718	85.68	100.00	539,512	995,741
GRVFV-6	OR787589	6727	MT084814	6718	96.53	100.00	400,088	477,244

**Table 6:** Nucleotide and amino acid similarities (%) between Czech and Italian sequences of GFkV.

Italian NC_003347	**5’UTR**	**Rep**		**CP**		**ORF3**		**ORF4**		**3′UTR**
	nt	nt	aa	nt	aa	nt	aa	nt	aa	nt
Czech OR701334	91.7	91.4	95.8	95.5	99.1	94.4	88.3	94.3	87.2	100

## Data Availability

The virus genomic sequences obtained in the present work have been deposited in the GenBank database of the National Center for Biotechnology Information (NCBI) under accession numbers OR701334, OR787584-OR787589, and OR826218-OR826260. Further data that support the findings of this study are available from the corresponding author upon request.

## Supplementary Materials

The following supporting information can be downloaded at: https://www.mdpi.com/article/10.3390/v16030343/s1. Table S1: List of reference sequences used for mapping; Table S2: List of primers used for virus detection; Table S3: List of accession numbers used for phylogenetic studies; Table S4: Recombination events detected in each dataset; Table S5: Primers for verification of newly obtained full-length sequences of viruses from the family *Tymoviridae*; Figure S1: Pairwise identity matrix, determined using SDT v. 1.2 software and based on the nucleotides of the complete coding region of 19 grapevine red globe virus, using citrus virus C (MN879754) as an outgroup; Figure S2: Pairwise identity matrix, determined using SDT v. 1.2 software and based on the nucleotides of the complete coding region of 57 grapevine rupestris vein feathering virus, using sequence of grapevine Syrah virus-1 (MZ440710) as an outgroup; Figure S3: Pairwise identity matrix, determined using SDT v. 1.2 software and based on the nucleotides of the complete coding region of 30 grapevine Syrah virus-1, using grapevine rupestris vein feathering virus (MZ451101) as an outgroup.
