# Supplementary material for: Multiple Infections with Viruses of the Family Tymoviridae in Czech Grapevines
Source: Viruses. 2024 Feb 23;16(3):343. doi: 10.3390/v16030343 (PMC10975331; doi:10.3390/v16030343)
Supplement: Supplementary file 1 [file viruses-16-00343-s001.zip › Figures S1-S3.pdf]

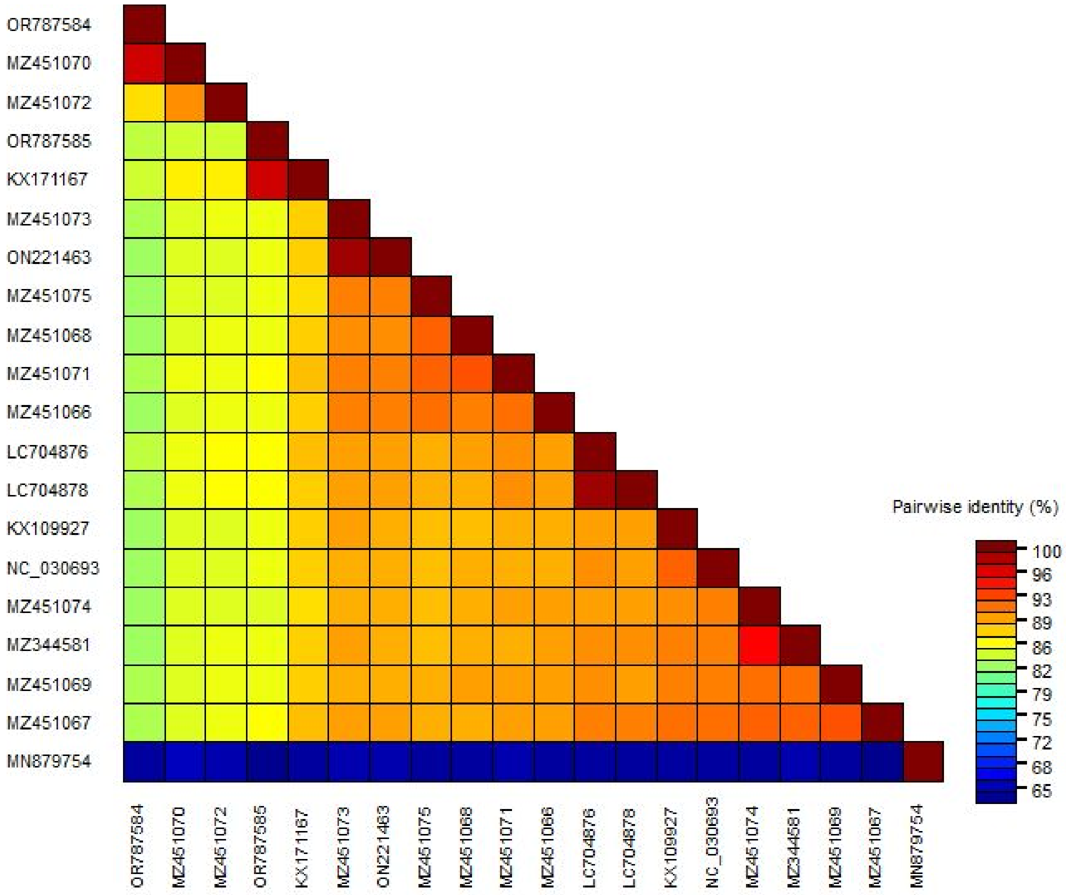

Figure S1 Pairwise identity matrix, determined using SDT v. 1.2 software and based on the nucleotides of the complete coding region of 19 grapevine red globe virus, using citrus virus C (MN879754) as an outgroup.

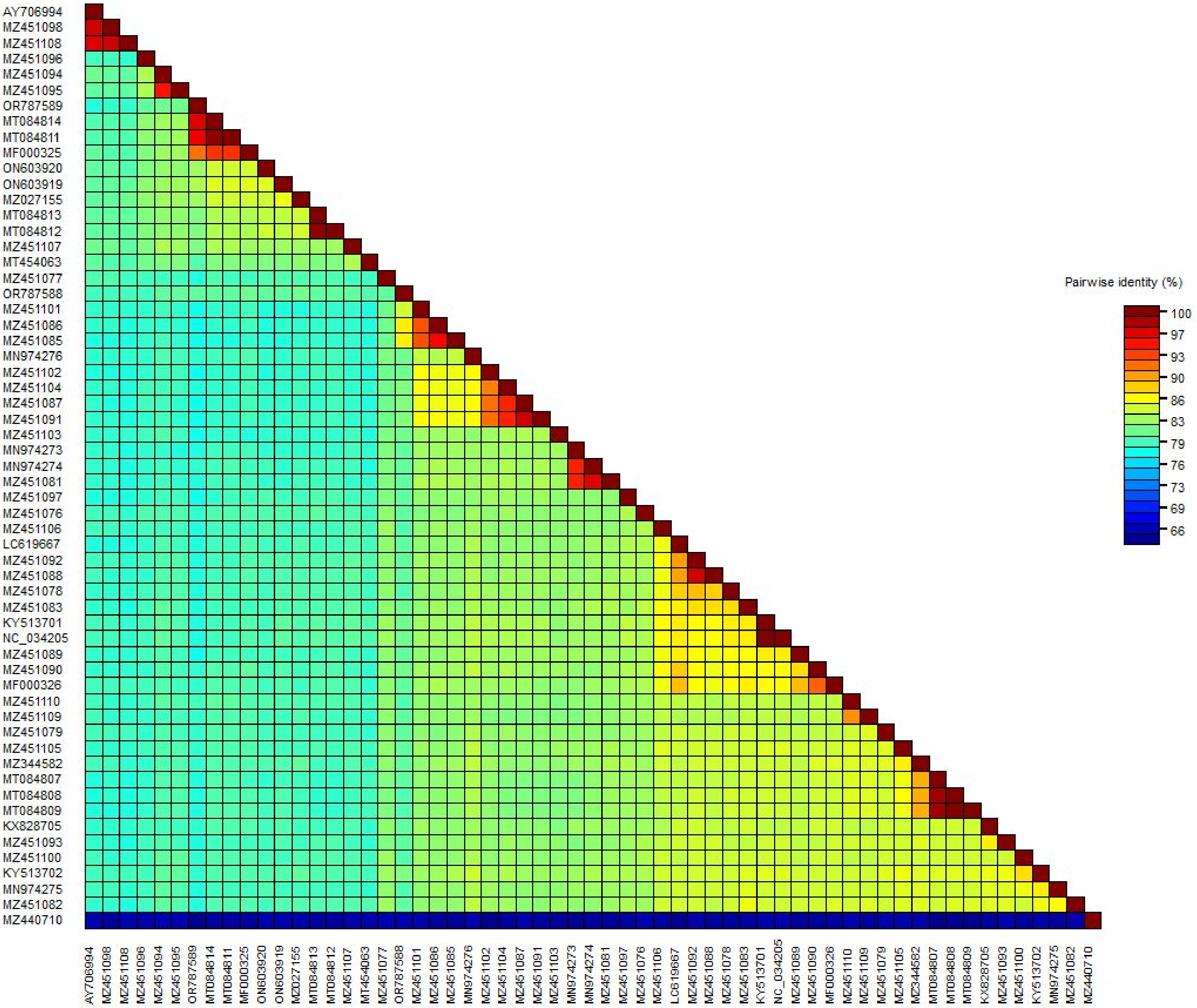

Figure S2 Pairwise identity matrix, determined using SDT v. 1.2 software and based on the nucleotides of the complete coding region of 57 grapevine rupestris vein feathering virus, using sequence of grapevine Syrah virus-1 (MZ440710) as an outgroup.

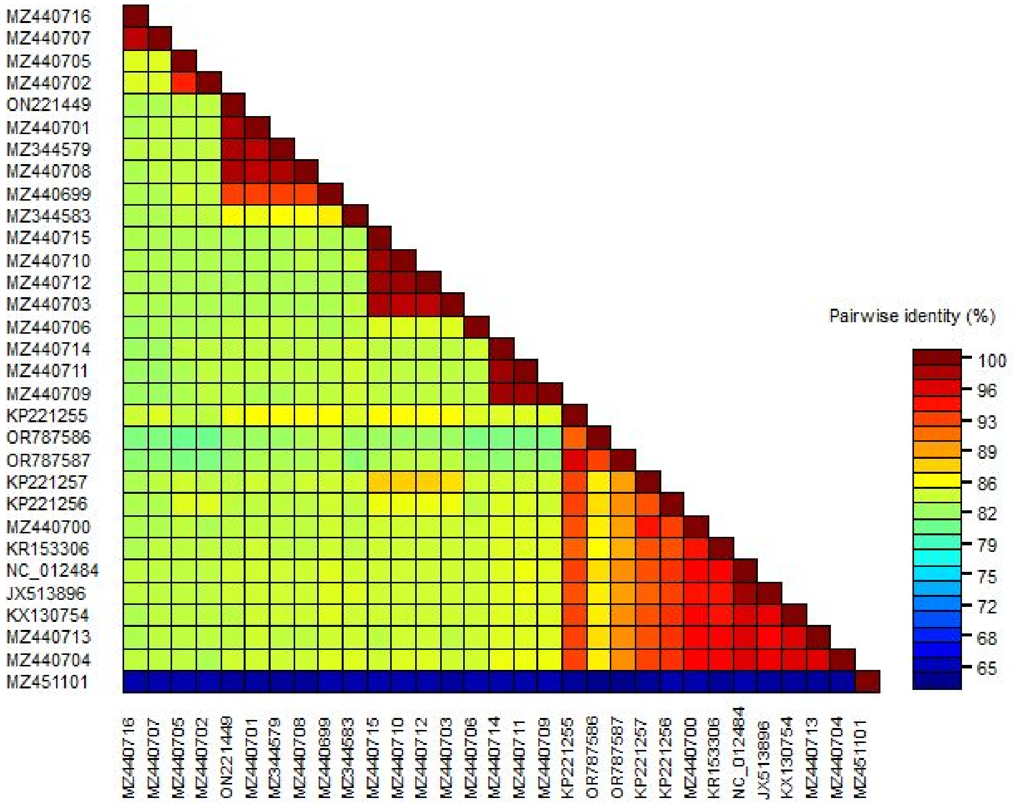

Figure S3 Pairwise identity matrix, determined using SDT v. 1.2 software and based on the nucleotides of the complete coding region of 30 grapevine Syrah virus-1, using grapevine rupestris vein feathering virus (MZ451101) as an outgroup.
