# Supplementary material for: Multiple Infections with Viruses of the Family Tymoviridae in Czech Grapevines
Source: Viruses. 2024 Feb 23;16(3):343. doi: 10.3390/v16030343 (PMC10975331; doi:10.3390/v16030343)
Supplement: Supplementary file 1 [file viruses-16-00343-s001.zip › Table S5 Primers for confirming tymovirus sequences.pdf]

Table S5: Primers for verification of newly obtained full-length sequences of viruses from the family *Tymoviridae*

Legend: first letter (F or R) indicates direction of primer (forward or reverse)

Next numbers indicate position in the virus genome.

GRGV OR787584

F6_OR787584: CTCAACCAGCCTCATCTCGA

R703_OR787584: AGGTTGGTCTCGATGGTCCT

F518_OR787584: CAAGGATACCATAGCGGCCC

R1430_OR787584: CTTCTGACTTTCGGTGCGGA

F1233_OR787584: GAAGCCCCCAAGAAGGACTC

R2107_OR787584: ACAGGGAGATCCTCAGGGAC

F1949_OR787584: CGCCTACCACCAGTACTTCC

R2830_OR787584: GTGAGGTGGTCAGTGGTGAG

F2634_OR787584: TCGTTACTCACCCGTGAACG

R3505_OR787584: TCGGCTTGGATTGTCAGGTC

F3323_OR787584: TAAAGTCGGCCTCCCCACTA

R4214_OR787584: GACGGGGGCATTGAAGAAGA

F4050_OR787584: CTCCCCCTCATCAAATCCCC

R4900_OR787584: AGAGTTTGGCAAGCCTTCCA

F4733_OR787584: CGTCAATGAATTCGCCCAGC

R5619_OR787584: GATTCTGAGGGAGCAGAGCC

F5419_OR787584: CCGAAGTTTCTCGCTACGGT

R6302_ OR787584: CTGGTGAGGGAGAGGAAGGG

F6126_OR787584: CATGGTGGCTCCTCCGAAG

R6841_OR787584: TTGAAGTGGGGCAAAGCAGA

GRGV OR787585

F1_OR787585: CTCAACCAGCCTCATCTCGA

R726_OR787585: GCAGAGTGGTGCCAGACATT

F514_OR787585: CAAGGACACCATAGCCGCC

R1426_OR787585: CTTCTGACTTTGGGTGCGGA

F1221_OR787585: CCACTCCTGAAGCTCCCAAG

R2099_OR787585: GAAGGTCCTCCGGGATGATG

F1941_OR787585: ATGATGCCGACCACCAGTAC

R2797_OR787585: CAGCTTGGTTGTCTCCGGAT

F2623_OR787585: CTCCTTCATGACCCGACAGC

R3502_OR787585: GGTGGGGTCAGATTGGATGG

F3345_OR787585: CCGAATGGAAGCGCAATCTC

R4194_OR787585: TTGAAGAAGACATCCCCGCA

F4019_OR787585: TTCCCCAAGCAACTCACAGG

R4896_OR787585: AGAGCAAGGGTCTGGCAAG

F4725_OR787585: TCAATGAGTTCGCCCAGCTC

R5608_OR787585: ATTCTCGGGGAGTAGGGTCC

F5413_OR787585: GGTCTCTCGCCACGGTTTAT

R6318_OR787585: ATTGGAGGACCGAGTAGGCA

F6120_OR787585: GTAGCCCCTCCGAAGCTTC

R6819_OR787585: GGCCAAGCAGAGACTGTGAA

GSyV-1 OR787586

F12_OR787586: CTGTCTCAACTGCTCTCGCA

R535_OR787586: CAAACCGTTGGAGGACAGGG

F334_OR787586: GTCAACATCCCCATCACCGT

R1251_OR787586: GTCGAACACTGTGCAAGGGA

F1059_OR787586: CCTCTACCTCTCCGTCACCA

R1948_OR787586: CTACGAAGCGGGGTTGTCTC

F1746_OR787586: CCTGAGGCGCTTCTTCTCC

R2640_OR787586: AGCAATGTCATCTTCGCGGA

F2430_OR787586: ATTCTTCTACCTGCACCCGG

R3339_OR787586: CAGACAACCCCTTGGCATCT

F3160_OR787586: CGCGCATTCAAGCACTTCAA

R4046_OR787586: TCACGAAGAACGATGGAGGC

F3863_OR787586: AAGTCGACCTTCGTGCTCTC

R4724_OR787586: AGAGTTTGGCAAGCCTTCCA

F4530_OR787586: TGAAGCTCTTTTCGCGGAGT

R5427_OR787586: GAGACTGTGCCCAATGGAGA

F5259_OR787586: GAAGGAGAAGTCCCGCTACG

R6117_OR787586: CACGGTGTTGAAGGTCCAGA

F5960_OR787586: TCTTCACCGTTGATCCTGCC

R6477_OR787586: AACAAACAAACGGAAAAGAAAACA

GSyV-1 OR787587

F12_OR787587: CTGTCTCAACTGCTCTCGCA

R535_OR787587: CAAACCGTTGGAGGACAGGG

F334_OR787587: GTCAACATCCCCATCACCGT

R1251_OR787587: GTCGAACACTGTGCAAGGGA

F1031_OR787587: TCAAAACCACCACCATTCGC

R1948_OR787587: CTACGAAGCGGGGTTGTCTC

F1738_OR787587: TTCCCTTTTCTGAGGCGCTT

R2616_OR787587: GGAGTCCGGGAAGTAACAGC

F2440_OR787587: CTTCACCCCGCTGATTGGAT

R3339_OR787587: CAGACAACCCCTTGGCATCT

F3160_OR787587: CGCGCATTCAAGCACTTCAA

R4046_OR787587: TCACGAAGAACGATGGAGGC

F3863_OR787587: AAGTCGACCTTCGTGCTCTC

R4726_OR787587: CGAGGGTTTGGCAAGCTTTC

F4548_OR787587: GTGCAGAGCTCTCAACGAGT

R5427_OR787587: GAGACTGTGCCCAATGGAGA

F5259_OR787587: GAAGGAGAAGTCCCGCTACG

R6123_OR787587: AGGCTCCACAGTGTTGAAGG

F5960_OR787587: TCTTCACCGTTGATCCTGCC

R6478_OR787587: AACAAACAAACGGAAAAGAAAACA

GRVFV OR787588

F19_OR787588: AAGTGCACCTTGACTCTAGC

R603_OR787588: GGAAATGGGAACTGGCTCCA

F419_OR787588: TCCCTTCTCTCTTCCGTCGT

R1285_OR787588: CGAGGCGATAGATGAGCTGG

F1095_OR787588: TTGGTTCATGCACGACTCCA

R1975_OR787588: CAAGGCTGAACCGAGGGTAG

F1810_OR787588: GCCTCCGCTTCTCTTATGCT

R2696_OR787588: GCACCAGAGGAGTCGATCAT

F2512_OR787588: GAAGCCAAGCCTCCTGTTCT

R3374_OR787588: ATGTGGATGAGCGAAACGGT

F3197_OR787588: ACTCTTACACCTCGCACGTG

R4077_OR787588: GGACAGATCGCCAGTGAACA

F3910_OR787588: AACGATTGTGGCTTTGCTGC

R4790_OR787588: AAGATGCGAACAGCTGACCA

F4585_OR787588: GAGATTCTGGGGTCCCTCCT

R5472_OR787588: GTCGAGGCACACCATGAGTT

F5283_OR787588: TGGCGATGACTCCCTTCTTG

R6205_OR787588: AGACGCAATGGAGAGTGACG

F6018_OR787588: CATCCCCTTCCAGTGGGTTG

R6578_OR787588: TAAAGCACACCAAGGTCGCT

GRVFV OR787589

F11_OR787589: AGCTCTCGCTACACTCAAGT

R656_OR787589: GAGAGCAGGTCGGTAAAGCC

F455_OR787589: CTCTCTCCTCGCCGACTTCC

R1377_OR787589: AGCTCTCCAGGCGAGTTCTA

F1185_OR787589: GAGTCCTCCTTCACCGACCT

R2077_OR787589: CGACGGGGTAAGAATCGCTT

F1874_OR787589: CTACCTCATTGGATCCCCGC

R2764_OR787589: CGAGAAGGGAAAGAACCGCT

F2557_OR787589: CTGATGACCTTCTGAGCCCC

R3442_OR787589: TAATTGCGACAGGGCTTGGT

F3257_OR787589: CGGCGTCCTTGCTAACCTAG

R4158_OR787589: AAGGGAAGAGAGAGCGGAGT

F3956_OR787589: CTACAATCACCCAGCCTGCA

R4875_OR787589: TAGCTTGGGTCTTGCTGCTC

F4656_OR787589: CTCCTCAAGGCCTCCATCG

R5550_OR787589: AGTAACCACAGAACTCGCCG

F5374_OR787589: ATTTCGCCGTTCTGCATCTG

R6244_OR787589: TTTTTCAACGTGGTGGAGGC

F6057_OR787589: CCCACTTCTGGTCGTCCTTC

R6713_OR787589: GCACACCAAGGTCACTTTACG
